# Temporal weights in loudness: Investigation of the effects of background noise and sound level

**DOI:** 10.1371/journal.pone.0223075

**Published:** 2019-11-05

**Authors:** Alexander Fischenich, Jan Hots, Jesko Verhey, Daniel Oberfeld

**Affiliations:** 1 Department of Psychology, Johannes Gutenberg-Universität Mainz, Mainz, Germany; 2 Department of Experimental Audiology, Otto von Guericke University Magdeburg, Magdeburg, Germany; Nottingham Trent University, UNITED KINGDOM

## Abstract

Previous research has consistently shown that for sounds varying in intensity over time, the beginning of the sound is of higher importance for the perception of loudness than later parts (primacy effect). However, in all previous studies, the target sounds were presented in quiet, and at a fixed average sound level. In the present study, temporal loudness weights for a time-varying narrowband noise were investigated in the presence of a continuous bandpass-filtered background noise and the average sound levels of the target stimuli were varied across a range of 60 dB. Pronounced primacy effects were observed in all conditions and there were no significant differences between the temporal weights observed in the conditions in quiet and in background noise. Within the conditions in background noise, there was a significant effect of the sound level on the pattern of weights, which was mainly caused by a slight trend for increased weights at the end of the sounds (“recency effect”) in the condition with lower average level. No such effect was observed for the in-quiet conditions. Taken together, the observed primacy effect is largely independent of masking as well as of sound level. Compatible with this conclusion, the observed primacy effects in quiet and in background noise can be well described by an exponential decay function using parameters based on previous studies. Simulations using a model for the partial loudness of time-varying sounds in background noise showed that the model does not predict the observed temporal loudness weights.

## Introduction

Loudness is one of the fundamental aspects of auditory sensation and is important when it comes to the perception of our environment through the auditory channel. Extensive research on steady-state sounds has been done in the past and loudness models are available that account for a large proportion of the psychoacoustic data [1–4]. However, static loudness models do not seem to account for all aspects of the loudness of sounds that vary in level across time [5,6].

One reason may be that not all temporal parts of a sound are weighted equally when listeners judge loudness. Previous studies consistently show that the beginning of a time-varying sound is of higher importance for the perception of loudness than later temporal parts [7–9], which has been referred to as a *primacy effect* (for a review see [10]). The primacy effect can be described by an exponential decay function with a time-constant of about 200–300 ms [10,11]. The temporal weight at the beginning of the sound is 4 to 5 times higher than the asymptotic weight, and the weight assigned to a temporal portion of a sound is the integral of this function over the segment duration [11]. In addition to the primacy effect, some studies also found higher perceptual weights assigned to the end of the sounds, commonly referred to as a recency effect [8,9].

Oberfeld and Plank [7] suggested several potential explanations of the primacy effect. First, the primacy effect might originate from an attention orientation response [12–14] to the sound onset, in the sense that the sound onset captures the attention [15]. However, their data did not support this explanation. They presented sounds starting with a gradual increase in level across the first few hundred milliseconds ("fade-in"). This should have reduced the perceived abruptness of the onset and, as a consequence, the capture of attention [16,17]. However, the data in [7] showed a delayed primacy effect in the sense of a high weight on the first unattenuated temporal segment. Another possible source of the primacy effect might be the response characteristic of auditory nerve (AN) neurons which tend to show a peak in the firing rate at the onset of a sound [18–21]. However, because the activity of the auditory pathway involves different types of neurons and efferent as well as afferent loops, a precise model of the auditory nerve would be required to evaluate to which extent the observed primacy effect can be explained on this basis.

Here, we investigate two factors that could potentially have an effect on both an attention orienting response and on the response of auditory nerve neurons. The first factor is the presence or absence of a continuous background noise, and the second factor is the level of the target sound.

Concerning the first factor, all previous experiments measuring temporal loudness weights presented sounds in quiet. This is a rather artificial condition since in a real environment, a target sound (we are listening to) is usually embedded in a background made of other sounds. The aim of the present study was thus to investigate the influence of a continuous *background noise* on the occurrence of the primacy and recency effects. The target sounds were presented in quiet as well as in a background noise. The presence of the continuous background noise could be expected to reduce the capture of attention by the target sound onset, because it makes the onset of this sound less abrupt or less surprising. Although we are not aware of relevant studies using auditory distractors, in the visual domain attentional capture appears to occur only for abrupt onsets of visual distractors [16,17]. Therefore, if the primacy effect was due to attentional capture to the target sound onset, it should be reduced for sounds that are presented in continuous background noise. In addition, responses of auditory nerve neurons are sensitive to background stimuli in the way that in the presence of a background noise the initial peak becomes smaller compared to the steady-state rate [22]. Thus, if the primacy effect was related to the initial peak in the AN firing rate, we assume that its size should be reduced in the presence of a background noise.

Concerning the second factor (*target level*), several studies investigated the effect of the relative level of temporal target components *within* a sound on the perceptual weights, showing that target components with a higher relative level receive a higher weight ("loudness dominance"; e.g., [7,23]). However, no previous study systematically tested for an effect of the average target level on the temporal loudness weights. Therefore, in the present study we measured temporal loudness weights for several different average sound pressure levels of the target sounds. A variation of the average target level might again affect the capture of attention to the sound onset, in the sense that the onset of a target sound presented only a few decibels above the detection threshold might appear less abrupt than the onset of a target sound with much higher level [16,17]. This leads to the prediction that if the primacy effect is related to attentional capture, more pronounced primacy effects should be found in the conditions with a higher target level, compared to target sounds presented just above the detection threshold. However, we are not aware of studies investigating the effect of the intensity of auditory or visual distractors on attentional capture.

In addition, the sound level has an effect on the responses of auditory nerve neurons, which show an increase in the initial peak of the firing rate relative to the steady-state response with increasing sound level [18,20]. Therefore, if the primacy effect was due to the initial peak in the firing rate of auditory nerve neurons, we hypothesize that it should be more pronounced at higher target sound levels.

To investigate the influence of both target level and background noise simultaneously, some of the experimental conditions presented the same average target sound pressure level in quiet and in background noise. In addition, a condition in quiet was compared to a condition in background noise at the same level above threshold, i.e., the same sensation level. This made it possible to test whether or not the primacy effect depends on the level or the signal-to-noise-ratio, which was not investigated before.

## Method

### Listeners

Eight normal-hearing listeners (7 female, 1 male, age 18–26 years) participated in this study. The experiment was conducted according to the principles expressed in the Declaration of Helsinki. All listeners participated voluntarily and provided informed written consent, after the topic of the study and potential risks had been explained to them. They were uninformed about the experimental hypotheses. The Ethics Committee of the Institute of Psychology of the Johannes Gutenberg-Universität Mainz approved the study (reference number 2016-JGU-psychEK-002). Hearing thresholds were measured by Békésy audiometry with pulsed 270-ms pure tones. All participants showed thresholds lower than or equal to 15 dB HL in the frequency range between 125 Hz and 8 kHz in both ears. All participants were students from Johannes Gutenberg-Universität Mainz and received partial course credit for their participation.

### Stimuli and apparatus

The stimuli were level-fluctuating noises consisting of ten bandpass-filtered low-noise noise segments. Bandpass-filtered noise was generated by transforming a broadband Gaussian noise into the frequency domain via a Fast Fourier Transform (FFT), setting the frequency components to zero outside the desired passband, and transforming it back to the time domain via an inverse FFT. The lower and upper cutoff frequencies were 770 Hz (7 Bark) and 1270 Hz (9 Bark), respectively. To reduce the intrinsic level fluctuations of the noise, the stimulus was multiplied by the inverse of its Hilbert envelope and then again bandlimited in the frequency domain, as described above. This multiplication and bandlimiting was repeated once. The resulting noise is commonly referred to as low-noise noise (LNN). This procedure to generate LNN is the second method proposed in Kohlrausch et al. [24]. Each noise segment had a duration of 120 ms including 20-ms cos^2^ on- and off-ramps. The segments were presented with a temporal overlap of 20 ms, resulting in a total stimulus duration of 1020 ms. Level fluctuations were created by drawing each segment’s sound pressure level independently and at random from a normal distribution on each trial.

In some conditions, the stimuli were presented in continuous background noise. The background noise was a Gaussian bandpass noise that was filtered with a 3^rd^-order Butterworth filter with cutoff frequencies of 300 Hz (3 Bark) and 2320 Hz (13 Bark), presented diotically with a sound pressure level of 50 dB SPL.

All sounds were generated digitally with a sampling frequency of 44.1 kHz, D/A-converted by an RME ADI/S with 24 bit resolution, attenuated by a TDT PA5 programmable attenuator, buffered by a TDT HB7 headphone buffer, and presented diotically via Sennheiser HDA 200 circumaural headphones. The reproducing system was calibrated according to IEC 60318–1:1998 [25] and free-field equalized as specified in ISO 389–8 [26]. Participants were tested in a double-walled sound-insulated chamber. Instructions were presented on a computer screen.

### Procedure

#### Measurement of detection thresholds

In order to ensure the same audibility of the target sounds in the loudness judgment task, for each participant, the individual detection thresholds for a single 120-ms LNN-segment (including 20-ms on- and off ramps) were determined in quiet and in the presence of the continuous background noise. Thresholds were measured using an adaptive two-interval, two-alternative forced-choice task [27]. On each trial, there were two observation intervals. In one of the two observation intervals (selected randomly), the signal (120-ms LNN-segment) was presented. The other interval contained no signal. Listeners selected the interval containing the signal. The initial signal level was 45 dB SPL in the condition in background noise and 20 dB SPL in the in-quiet condition. The level of the signal was adjusted by a three-down, one-up adaptive rule. The step size was 8 dB until the third reversal, and 2 dB for the remaining six reversals. The arithmetic mean of the signal levels at the final six reversals was taken as the detection threshold, corresponding to 79.4% correct. For each listener, a minimum of three adaptive blocks was presented for each of the two conditions (in quiet and in background noise). A block was discarded if the standard deviation of the signal levels at the six final reversals was greater than 6 dB. In the following, the average detection threshold in quiet is denoted *L*_th_quiet_, and the detection threshold in background noise is denoted *L*_th_BGN_. Resulting thresholds for the eight listeners are shown in Table 1. Across the eight listeners, the mean threshold in quiet was 3.6 dB SPL (SD = 5.9 dB, min = −3.6 dB SPL, max = 13.4 dB SPL), and under masking 43.2 dB SPL (SD = 3.1 dB, min = 40.2 dB SPL, max = 50.0 dB SPL).

**Table 1:** Detection thresholds and mean target sound pressure levels for the eight listeners. Values are reported in dB SPL. For the calculation of the three different target sound pressure levels (*L*_SLquiet7.5_, *L*_SLBGN7.5_ and *L*_SLBGN30_) see section *Measurement of temporal loudness weights*.

Listener	*L*_th_quiet_	*L*_th_BGN_	*L*_SLquiet7.5_	*L*_SLBGN7.5_	*L*_SLBGN30_
1	−3.6	41.2	3.9	48.7	71.2
2	2.3	41.9	9.8	49.4	71.9
3	7.0	50.0	14.5	57.5	80.0
4	1.2	41.0	8.7	48.5	71.0
5	−2.0	43.1	5.5	50.6	73.1
6	13.4	43.3	20.9	50.8	73.3
7	0.8	40.2	8.3	47.7	70.2
8	9.9	44.6	17.4	52.1	74.6

*L*_th_quiet_ = Threshold in quiet. *L*_th_BGN_ = Threshold in background noise.

*L*_SLquiet7.5_, *L*_SLBGN7.5_ & *L*_SLBGN30_ = Means of the target sound pressure level distributions.

#### Measurement of temporal loudness weights

To estimate temporal loudness weights, we used an established experimental paradigm from previous experiments (e.g., [7,10,11]). The target stimuli were level-fluctuating narrowband noises consisting of ten contiguous temporal segments. On each trial, the ten segment levels were selected by drawing each segment’s sound pressure level independently and at random from a normal distribution.

Temporal weights were measured for two mean segment levels in conditions with continuous background noise (termed BGN in the following), and for three mean segment levels in conditions without background noise (in quiet). In the first condition in background noise, the mean of the normal distribution from which the segment levels were drawn was 7.5 dB higher than the individual detection threshold for a single temporal segment in the continuous background noise (see previous subsection on the measurement of detection thresholds), *L*_SLBGN7.5_ = *L*_th_BGN_ + 7.5 dB. Thus, on average, the signal had a sensation level (SL) of 7.5 dB SL under masking, and this condition is referred to as BGN_SLBGN7.5_. In the second condition in background noise, the mean of the normal distribution was 30 dB higher than the individual detection threshold in the background noise, *L*_SLBGN30_ = *L*_th_BGN_ + 30 dB. This condition is referred to as BGN_SLBGN30_. Two in-quiet conditions (i.e., without background noise) with the same mean sound pressure level of the segments as in the two conditions in background noise were presented: Quiet_SLBGN7.5_ used the same mean segment level as BGN_SLBGN7.5_, and the mean segment level in Quiet_SLBGN30_ was the same as in BGN_SLBGN30_. In addition, an in-quiet condition with the mean segment level set to 7.5 dB SL (that is, 7.5 dB above the individual detection threshold for a single segment in quiet), *L*_SLquiet7.5_ = *L*_th_quiet_ + 7.5 dB, referred to as Quiet_SLquiet7.5_, was included in the experiment. Table 2 provides an overview of the five experimental conditions and the corresponding target levels. Table 1 shows the three different resulting mean target sound pressure levels for the eight listeners.

**Table 2:** Overview of the experimental conditions and the corresponding grand means of the target level distributions. Δ*L* specifies the difference in mean level between the two level distributions presented in the loudness-judgment task in each condition.

In background noise		In quiet	
Condition	Mean target level	Condition	Mean target level
BGN_SLBGN7.5_	*L*_SLBGN7.5_ = *L*_th_BGN_ + 7.5 dB	Quiet_SLBGN7.5_	*L*_SLBGN7.5_
	Δ*L* = 2.5 dB		Δ*L* = 2 dB
BGN_SLBGN30_	*L*_SLBGN30_ = *L*_th_BGN_ + 30 dB	Quiet_SLBGN30_	*L*_SLBGN30_
	Δ*L* = 2 dB		Δ*L* = 2 dB
		Quiet_SLquiet7.5_	*L*_SLquiet7.5_ = *L*_th_quiet_ +7.5 dB
			Δ*L* = 2.5 dB

**Fig 1:**
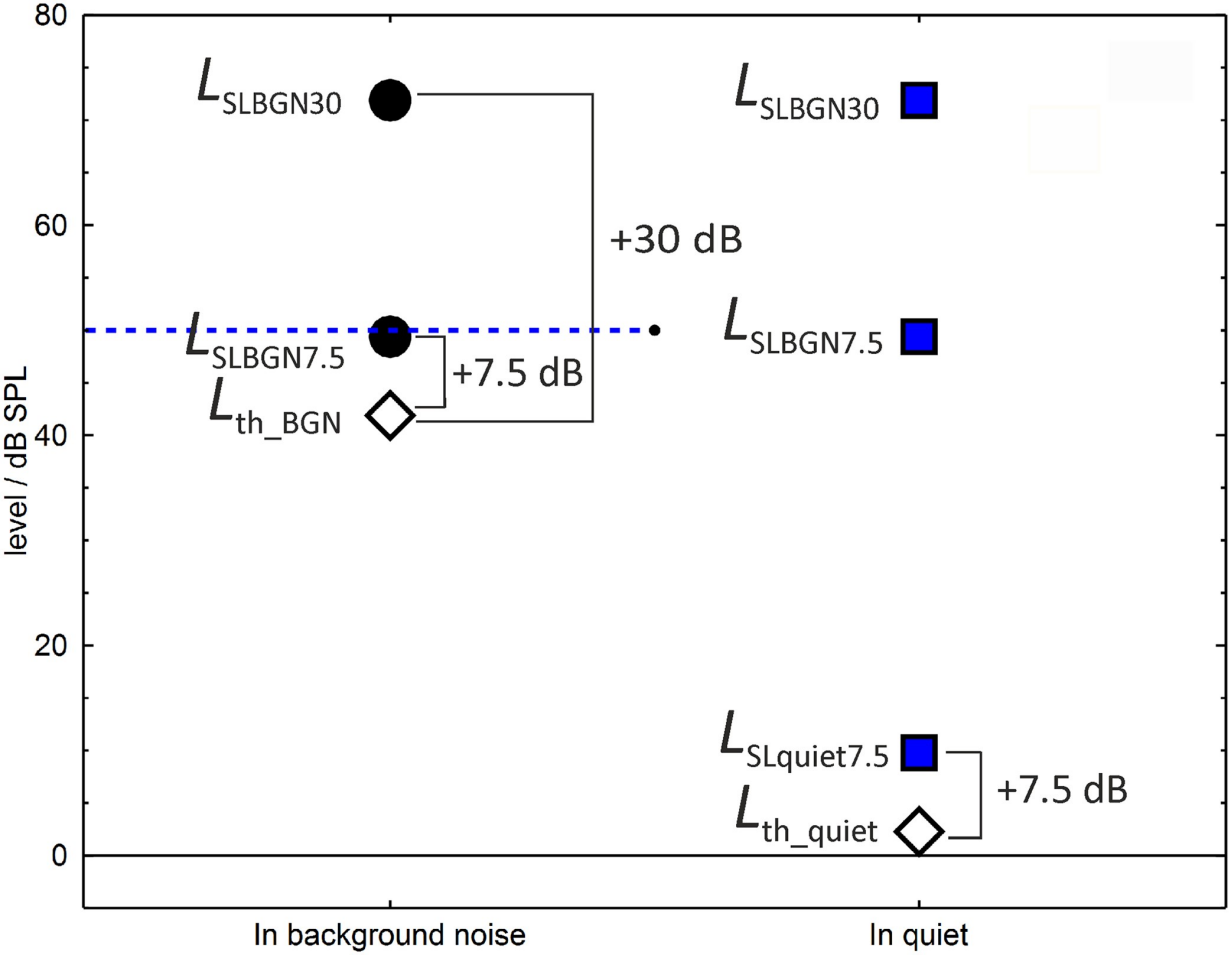
Detection thresholds and mean segment levels of the loudness judgment task, for one listener. The individual means of the level distributions in the loudness judgment task were determined by adding the indicated levels to the individual detection thresholds. The blue dashed line indicates the level of the continuous background noise in the conditions in background noise.

Fig 1 shows an example of the individual thresholds and mean target levels for a listener with a threshold in quiet of *L*_th_quiet_ = 2.3 dB SPL and a threshold in background noise of *L*_th_BGN_ = 41.9 dB SPL. The figure illustrates that we varied both the absolute sound pressure level of the target sounds and the level of the target sounds relative to the individual detection thresholds.

The standard deviation of each of the level distributions was *σ* = 2.5 dB. Unacceptably loud or soft segments were avoided by limiting the range of possible sound pressure levels to three standard deviations below and above the mean of the distribution.

**Fig 2:**
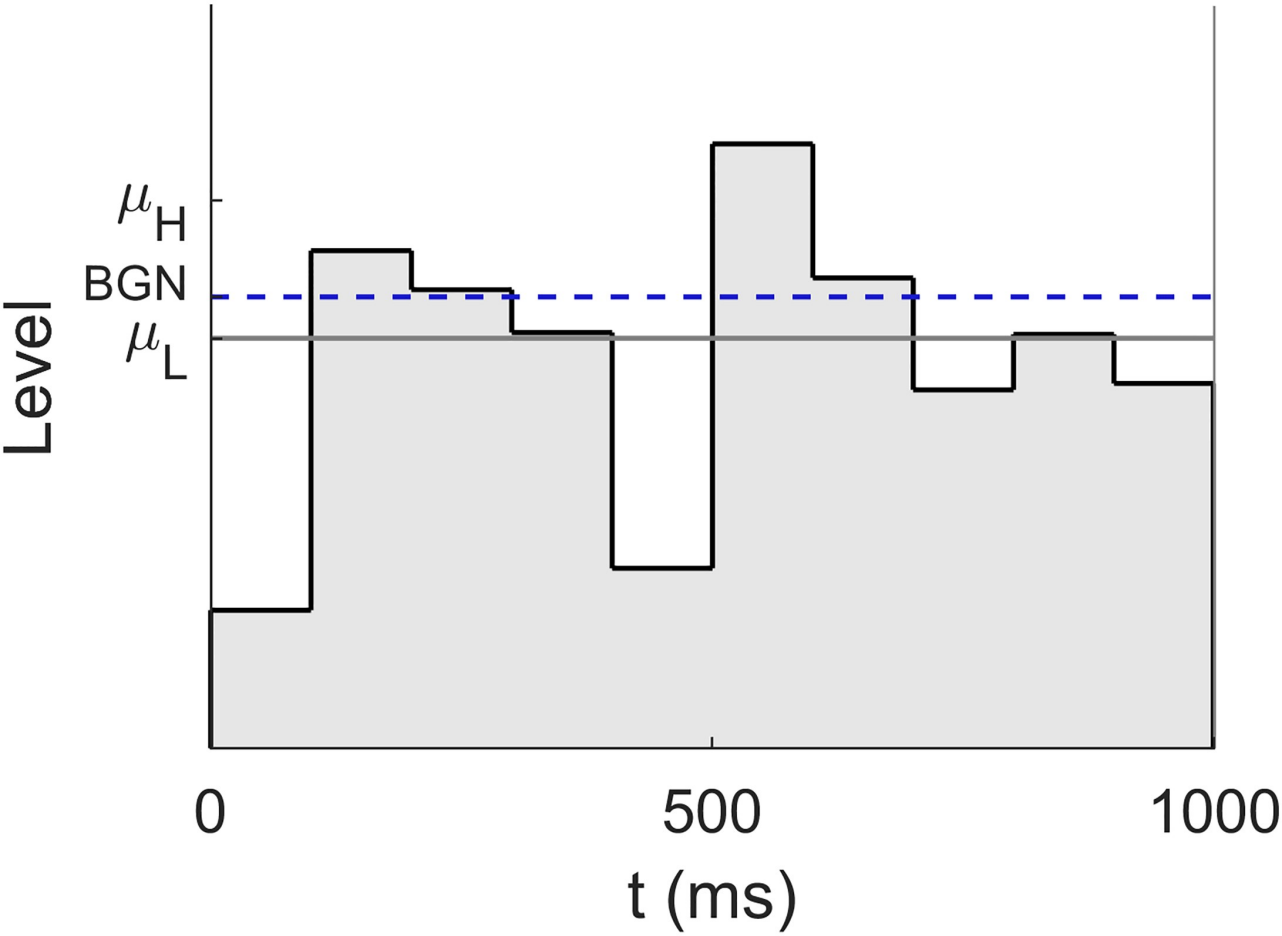
Schematic plot of the time-varying stimuli in background noise in condition BGN_SLBGN7.5_. In this example, all segment levels are drawn independently from the distribution with the lower mean (μ_L_ = *L*_SLBGN7.5_ − Δ*L*/2) and a standard deviation σ = 2.5 dB. The mean level is represented by the horizontal gray line. The dashed blue line indicates the level of the continuous background noise. Please note that on- and off-ramps are excluded in this depiction.

For each condition, a distribution with higher mean and a distribution with lower mean were created by adding or subtracting a value of Δ*L*/2 to the individual mean segment level in the corresponding condition, respectively. On each trial, there was a 0.5 probability of presenting a sound with segment levels drawn from the distribution with the higher or lower mean. For conditions BGN_SLBGN30_, Quiet_SLBGN 7.5_, and Quiet_SLBGN30_, Δ*L* was 2.0 dB, while for BGN_SLBGN7.5_ and Quiet_SLquiet7.5_, Δ*L* was 2.5 dB. These values were selected on the basis of pilot data in order to maintain a performance level of about 70% correct. Please note that the choice of a minimum SL of 7.5 dB in quiet as well as in background noise ensured that, despite the random level fluctuations, all segment levels were at or above the individual thresholds in virtually all of the trials. Fig 2 shows a fluctuating level profile of a sound drawn from the distribution with the lower mean in condition BGN_SLBGN7.5._

After each trial, listeners decided whether the presented target sound had been "louder" or "softer". They were instructed to use previous trials within an experimental block as a reference for their loudness judgments. The inter-trial interval was 1500 ms, with the restriction that the next trial never started before the response to the preceding trial had been given. Trial-by-trial feedback was provided during the first seven trials of each block so that the participants could easily adopt a decision criterion for the new experimental condition. Those trials were not considered for the data analysis. A response was classified as correct if the response (“softer”/”louder”) matched the mean of the distribution that the stimulus’ segment levels were drawn from. A summarizing feedback was provided each time 50 trials were completed. It contained the number of correct and false answers, percent correct, the number of trials from the distribution with higher mean and the distribution with lower mean, and the number of “louder” and “softer” responses.

The usual rule of thumb from previous experiments [10] was used concerning the number of trials necessary to obtain reliable weight estimates, and 100 trials per temporal segment were presented. Since the sounds in each condition contained ten temporal segments, 1000 trials were presented per condition, resulting in a total of 5000 trials per listener.

#### Sessions

Each listener participated in five experimental sessions, each containing 1000 trials of the loudness judgment task (200 per condition). Additionally, there was an initial session in which audiometric thresholds and detection thresholds in quiet and under masking were measured, and practice blocks of the loudness judgment task were presented for all of the five conditions. The practice blocks were excluded from data analysis. Within each session, sounds of the same condition were arranged into blocks of 200 trials. The order of conditions was chosen randomly. The duration of each session was approximately 60 minutes, including a mandatory pause of about 5 minutes.

### Data analysis

The perceptual weights representing the importance of the 10 temporal segments for the decision in the loudness judgment task were estimated from the trial-by-trial data via multiple logistic regression. The decision model assumed that the listener compares a weighted sum of the segment levels to a fixed decision criterion, and responds that the sound was of the "louder" type if the weighted sum exceeds the criterion (a detailed description of the decision model is provided by [7]). If the weighted sum is smaller than the criterion, it is assumed that the listener classifies the sound as "softer". In the data analysis, the binary responses ("louder" or "softer" type of sound) served as the dependent variable. The predictors (i.e., the ten segment levels) were entered simultaneously. The regression coefficients were taken as the decision weight estimates.

A separate logistic regression model was fitted for each combination of listener and condition. Since the *relative* contributions of the different temporal segments to the decision were of interest rather than the absolute magnitude of the regression coefficients, the ten regression coefficients were normalized for each fitted model such that the mean of their absolute values was 1.0. This resulted in a set of relative perceptual weights for each combination of listener and condition. We also fitted separate logistic regression models to trials in the first and last experimental session (session 2 & session 6), again for each combination of listener and condition separately, to investigate whether the temporal weighting changed during the course of the experiment.

A summary measure of the predictive power of a logistic regression model is the area under the Receiver Operating Characteristic (ROC) curve (for details see [9]). Areas of 0.5 and 1.0 correspond to chance performance and perfect performance of the model, respectively. Across the 40 logistic regression models that were fitted to the pooled trials from all experimental sessions, the area under the ROC curve ranged between 0.59 and 0.92 (*M* = 0.76, *SD* = 0.11), indicating on average reasonably good predictive power [28].

The individual normalized temporal weights estimated by the multiple logistic regressions were analyzed with repeated-measures analyses of variance (rmANOVAs) using a univariate approach with Huynh-Feldt correction for the degrees of freedom [29]. The correction factor $\tilde{\varepsilon }$ is reported, and partial η^2^ is reported as a measure of association strength. An α-level of .05 was used for all analyses.

## Results

**Fig 3:**
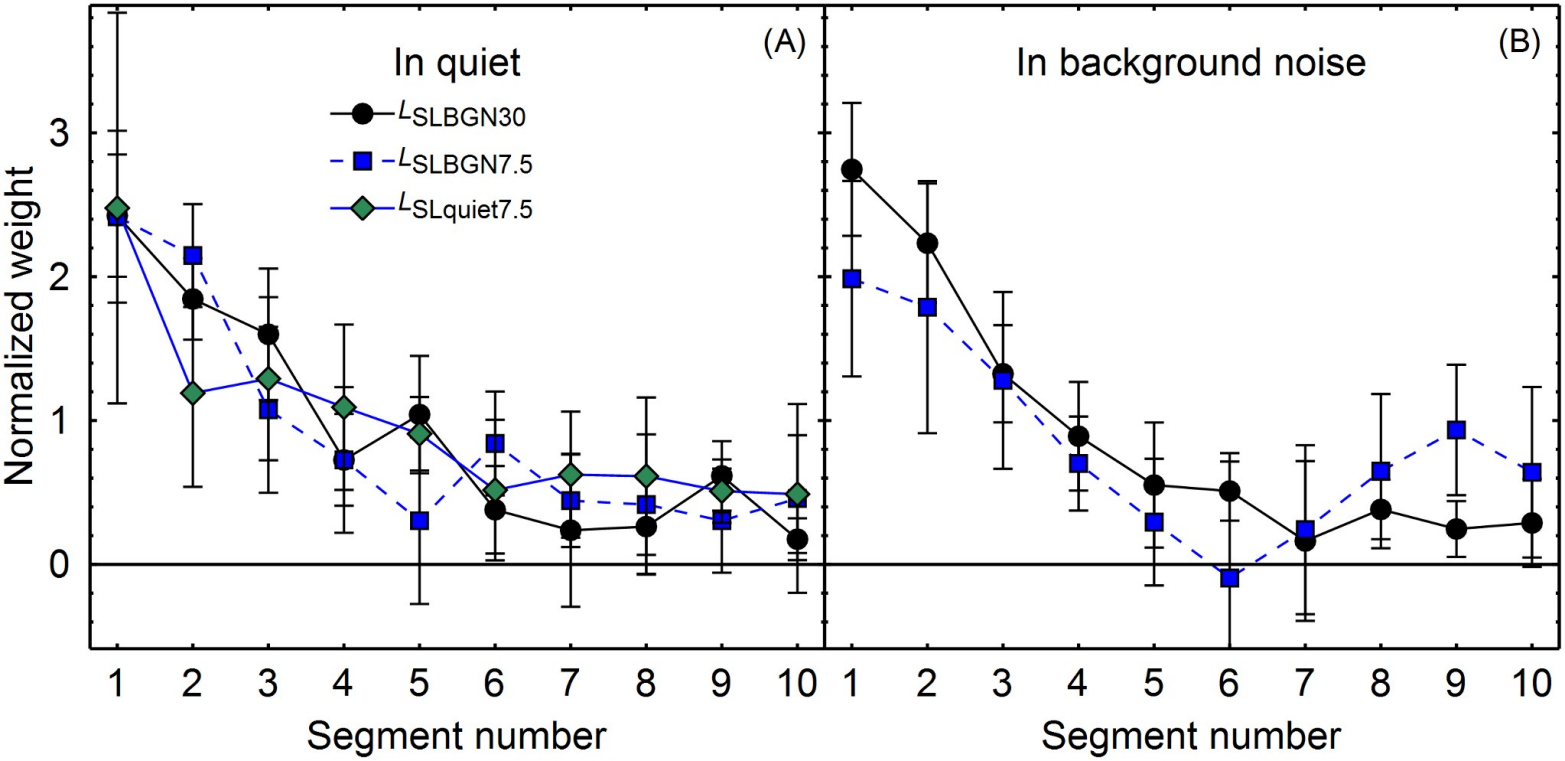
Mean normalized weights as a function of segment number. Panel A: in quiet. Panel B: in continuous background noise. The lines indicate the different mean segment levels (see Table 2). Black circles: *L*_SLBGN30_. Blue squares: *L*_SLBGN7.5_. Green diamonds: *L*_SLquiet7.5_. Error bars show 95% confidence intervals (CIs).

Fig 3 shows the mean normalized temporal weights in the five conditions. In each condition, a clear primacy effect was observed, in the sense that the weight on the first segment was higher than the weights on the following segments. For the condition in background noise where the mean segment level was 7.5 dB higher than the detection threshold in background noise (BGN_SLBGN7.5_), the primacy effect was slightly weaker than in the other conditions, and the mean weights on the final three segments were slightly higher than the weights for the middle segment, indicating a (weak) recency effect. Apart from this, the patterns of weights in the five conditions were similar.

An rmANOVA with the within-subjects factors condition (BGN_SLBGN30_, BGN_SLBGN7.5_, Quiet_SLBGN30_, Quiet_SLBGN7.5_, and Quiet_SLquiet7.5_) and segment number (1–10) was conducted. There was a significant effect of segment number, *F*(9, 63) = 29.95, $\tilde{\varepsilon }$ = .494, *p* < .001, $\eta_{p}^{2}$ = .811, confirming that the weights differed between the ten segments. The condition × segment number interaction was not significant, *F*(36, 252) = 1.91, $\tilde{\varepsilon }$ = .449, *p* = .058, $\eta_{p}^{2}$ = .214, which indicates that masking and signal level had no significant effect on the pattern of temporal weights. To evaluate whether listeners adopted a decisional strategy during the experiment that eventually lead to the uniform weights across the different conditions, we also analyzed the weights from the logistic regressions that were fitted to the first and the last experimental session for each combination of listener and condition separately. An rmANOVA with the within-subjects factors condition (BGN_SLBGN30_, BGN_SLBGN7.5_, Quiet_SLBGN30_, Quiet_SLBGN7.5_, and Quiet_SLquiet7.5_), segment number (1–10) and session (2,6) showed neither a significant main effect of session nor an interaction with session and any of the other factors. The relevant segment number × session and segment number × condition × session interactions both were not significant (*F*(9, 63) = 1.07, $\tilde{\varepsilon }$ = .855, *p* = .400, $\eta_{p}^{2}$ = 0.132; *F*(36, 252) = 1.14, $\tilde{\varepsilon }$ = .509, *p* = .317, $\eta_{p}^{2}$ = 0.141).

To quantify the magnitude and time course of the primacy effect, exponential decay functions were fitted to the mean weights at each of five conditions, as proposed in our previous work [10,11]. The weight assigned at the time *t* was assumed to be

$$w(t)\;=\;c(D_{r}\;∙e^{-\frac{t}{\tau }}\;+\;1),$$ (1)

where *t* = 0 corresponds to the sound onset, *c* is the asymptotic weight at *t → ∞*, *D*_*r*_ is the weight at sound onset (*t* = 0) relative to the asymptotic weight *w*(*∞*) = *c* (i.e., *D*_*r*_ is the "dynamic range" of the weights), and the time constant τ quantifies the time needed for the weight to decay to a value of 1/*e* of the weight range between *w*(0) and the asymptotic weight *c*. The weight assigned to a temporal segment with onset at *t*_on_ and duration *d* was assumed to be the integral of *w*(*t*) across the segment duration,

$$\bar{w}\left(t_{on},d\right)=\int_{t=t_{on}}^{t_{on}+d}w\left(t\right)dt.$$ (2)

**Fig 4:**
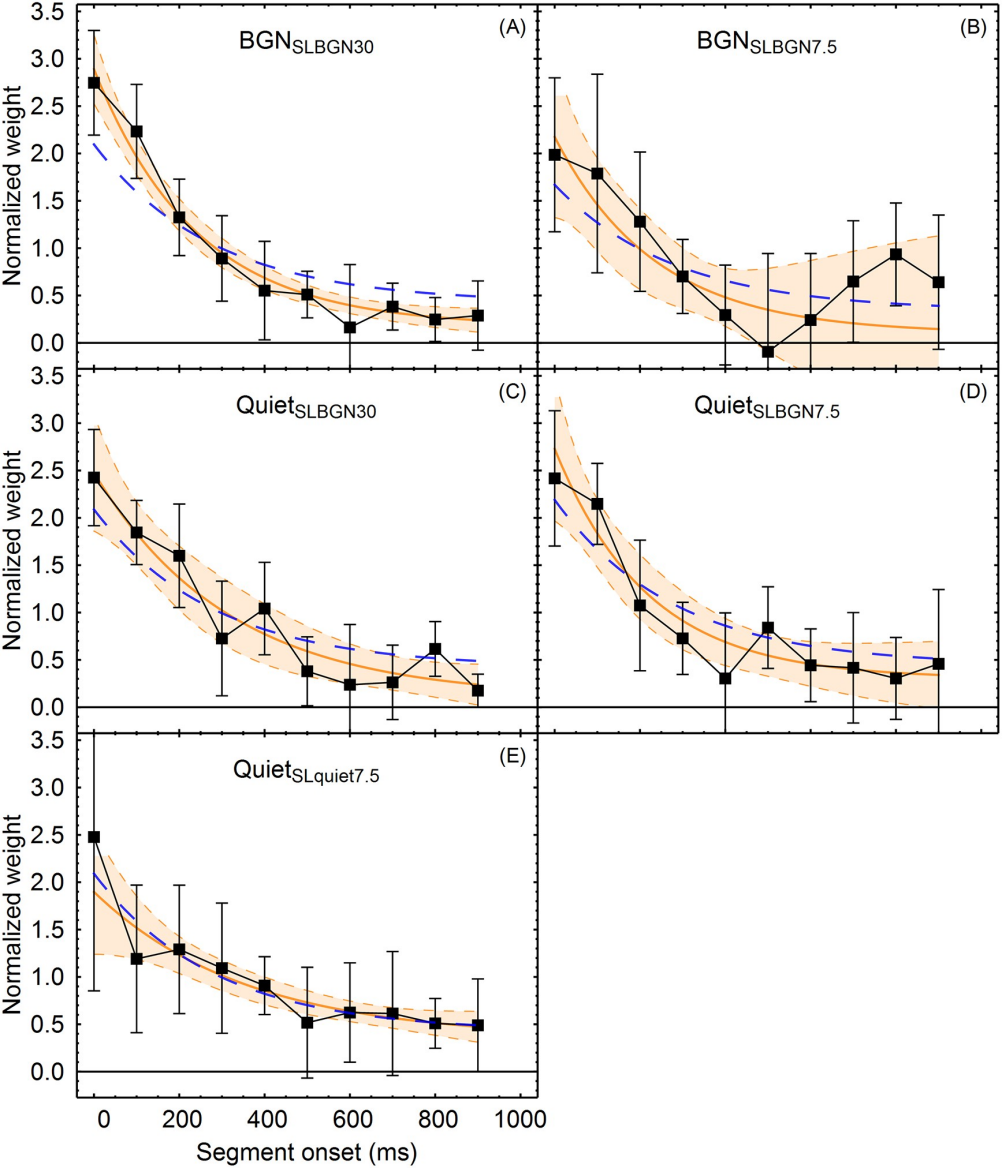
Fits of the exponential decay function as a function of segment onset and condition. For the fitted function see Eq 2. The panels A-E show the five different conditions. Panel A: BGN_SLBGN30_. Panel B: BGN_SLBGN7.5_. Panel C: Quiet_SLBGN30_. Panel D: Quiet_SLBGN7.5_. Panel E: Quiet_SLquiet7.5_. The solid orange line in each panel indicates the exponential decay function that was fitted to the data for this sound duration. Note that for condition BGN_SLBGN7.5_ (panel D), only the first seven segments (onsets between 0 and 500 ms) were used for fitting the decay function, due to the small recency effect observed in this condition. The orange shaded areas represent 95% confidence bands for the mean. The dashed blue line shows the average decay function based on previous experiments (Eq 2), using the average values of τ = 280 ms and *D*_r_ = 4.70 reported by Oberfeld et al. [11]. Error bars show ±1 standard error of the mean (SEM).

The function $\bar{w}(t_{on},d)\;$ was fitted to the mean weights in each experimental condition separately, using the Mathematica function *NonlinearModelFit*, with the weight for a given data point *w*_i_ proportional to $1/SD_{w_{i}}^{2}$, where $SD_{w_{i}}^{2}$ is the variance of the eight individual estimated weights for segment *i*. Since only the time course of the primacy effect was of interest (and not potential recency effects), the function was fitted only to the first seven weights at the BGN_SLBGN7.5_ condition. Here, the remaining mean weights descriptively showed a weak recency effect. Fig 4 shows the fitted functions, together with the average decay function estimated on the basis of previous experiments [10]. The estimated parameters of the fitted decay functions are shown in Table 3. The exponential decay function provided an excellent fit to the mean weights in all five conditions (*R*^2^ ≥ 0.96). However, the standard errors of the estimated parameters in some of the conditions were larger than in the previous experiments [10]. This was particularly pronounced for the BGN_SLBGN7.5_ condition, where only 7 of the 10 segment weights were included in the fitting procedure due to the small recency effect. The size of the time constant τ was comparable to previous studies in which the estimated τ varied between 180 ms and 300 ms [10]. In the present data, the estimated τ was about 240 ms for the conditions in background noise, whereas for the conditions in quiet, τ varied between 217 and 355 ms. Previous studies showed a dynamic range *D*_*r*_ of about 4.70 for comparably long sound durations [10,11]. In the current study, a similar dynamic range (*D*_*r*_ = 5.05) was only found for condition Quiet_SLquiet7.5_, whereas all other conditions showed higher dynamic ranges with *D*_*r*_ between 10 and 27.48.

**Table 3:** Fits of an exponential decay function (Eq 2) to the observed primacy effects in the five different conditions. The estimated parameters τ, D_r_, and c are displayed, together with the standard error of the estimate, the p-value for a test of the estimated parameter against 0, the 95% CI, R^2^ for the fitted function, and R^2^_avg_ for the decay function with average parameters (see text).

Condition	Parameter	Estimate	SE	*p*	95% CI lower	95% CI upper	*R*^2^	*R*^2^_*avg*_
BGN_SLBGN7.5_	τ	236.01	129.90	0.14	−124.65	596.67	0.96	0.90
	*D*_r_	25.42	117.04	0.84	−299.51	350.417		
	*c*	0.001	0.0045	0.84	−0.012	0.019		
BGN_SLBGN30_	τ	238.96	30.98	0.000	165.68	312.24	0.99	0.90
	*D*_r_	18.79	8.10	0.053	−0.35	37.94		
	*c*	0.0018	0.0008	0.056	−0.00006	0.0036		
Quiet_SLQuiet7.5_	τ	355.05	141.04	0.040	21.54	688.55	0.98	0.98
	*D*_r_	5.05	2.46	0.080	−0.78	10.87		
	*c*	0.0035	0.0018	0.085	−0.0006	0.0077		
Quiet_SLBGN7.5_	τ	217.72	69.66	0.017	53.00	382.44	0.96	0.93
	*D*_r_	10.00	6.27	0.15	−4.82	24.83		
	*c*	0.0030	0.0019	0.16	−0.0015	0.0075		
Quiet_SLBGN30_	τ	316.68	109.02	0.023	58.88	574.47	0.96	0.91
	*D*_r_	27.48	54.54	0.63	−101.50	156.46		
	*c*	0.001	0.0020	0.63	−0.0037	0.0058		

The dashed blue lines in Fig 4 show the average decay function with τ = 280 ms and *D*_*r*_ = 4.70 [11]. This average function was fitted to the mean weights in each condition of our experiment, using only the multiplicative constant *c* as a free parameter. As seen in Table 2, the goodness of fit of this mean decay function (*R*^2^_avg_) was only minimally lower than for the specific decay function in all conditions. Thus, the average decay function provides a reasonably good description of the primacy effect in quiet as well as in the conditions in background noise. This confirms that the presence or absence of a background noise and variations in target level have a negligible effect on the primacy effect.

## Discussion

### Temporal loudness weights in background noise

In the present study, the influence of the acoustic background on temporal loudness weights was assessed for the first time by presenting the target sound either in quiet or in a continuous background noise. The presence of the background noise did not result in significant changes in the pattern of temporal weights compared to conditions without background noise. This is in agreement with findings of Oberfeld and Plank [7]. They concluded from their results that the primacy effect does not stem from an attention orientation response due to the abrupt onset of the sound. This conclusion can also be drawn from the results of the present experiment, in which the background noise was continuously presented during each experimental block, which, as we assumed in the introduction, should have reduced a potential attention orientating response to the onset of the target sound, relative to an in-quiet condition because the abruptness of the onset of the stimulus is reduced [16,17]. However, this is just an assumption based on the above mentioned research on attention capture in the visual domain, while to our knowledge, auditory attention orienting in quiet and with background noise has not been compared so far.

The absence of an effect of the background noise on the temporal weights is surprising considering that background noise affects the neuronal responses at several stages of the auditory pathway [22].

In addition to effects of background noise, we also investigated effects of the average target level. In quiet, a variation in mean level across a range of about 60 dB (from 7.5 dB above the individual threshold in quiet to 30 dB above the detection threshold in a 50-dB SPL background noise; see Table 2) had no significant effect on the temporal weights in the in-quiet condition. In background noise, the primacy effect was descriptively slightly less pronounced at the lower sound level compared to the higher sound level. Also, descriptively a small recency effect was observed at the lower sound level. However, the temporal weights did not differ significantly between the five experimental conditions. Taken together, varying the average target level across a wide range resulted only in surprisingly small effects on the temporal loudness weights. This pattern is at odds with an explanation of the primacy effect in terms of an orienting response, because as we mentioned in the introduction, a lower mean target level should have made the target sound onset less abrupt [16,17]. Again, the absence of a significant effect of target level on the weights is surprising because the responses of neurons at many stages of the auditory pathway are affected by the sound level.

One explanation of the primacy effect, which has been suggested in Dittrich and Oberfeld [9] is that it might be caused in a memory system, because it resembles the serial position effects commonly found in memory tasks. However, in Oberfeld et al. [10] this assumption was not supported. They presented sounds consisting of 20 segments and varied the length of the sounds between 1.0 s and 10.0 s. The data indicated that the weight assigned to a specific temporal portion of the sound is determined by the time delay between sound onset and segment onset, rather than by the relative position of the segment within the sound (i.e., segment number). In contrast, in memory experiments, the serial position curve is determined by the relative temporal position of the items within the item lists, while the time delay between the onset of the list and the given item plays only a minor role [30].

Another potential explanation we are currently evaluating is whether the effects may stem from an "evidence integration" or "sequential sampling" process in which evidence for one of the two possible hypotheses (“louder” or “softer” type of sounds) is collected sequentially during each trial and the decision is made as soon as sufficient information has been accumulated, ignoring further evidence during the trial [31,32]. Some recent papers found primacy (and sometimes recency) effects in visual motion perception [32] or brightness perception [33], using time-varying stimuli as in our experiment. These temporal weighting patterns can be modeled by accumulator or drift diffusion models. The authors of the respective papers thus suggested that the temporal weights are not caused at an early, sensory stage, but rather at a later decisional stage. An interesting prediction from this hypothesis is that within a participant, the temporal weights applied for instance in a loudness judgment task for sounds varying in level across time as in the present paper, and in a brightness judgment task for visual stimuli varying in brightness across time should be similar, because both of them are due to a certain type of evidence integration at the decision stage. We are currently testing this hypothesis in an experiment where participants receive randomly interleaved loudness-judgment and brightness-judgment trials.

### Temporal weights predicted by a dynamic loudness model

To test if the observed loudness weights are predicted by an existing dynamic loudness model, the predictions of a model for the partial loudness of time-varying-sounds in background noise [34] were analyzed. The model, which we refer to as the time-varying partial loudness (TVPL) model, is based on the time-varying loudness (TVL) model by Glasberg and Moore [35]. The model was slightly modified by using the attack and decay time constants from the most current version of the TVL model [36]. An instantaneous partial loudness (IPL) is calculated from the predicted excitation patterns for signal and background noise [34]. The *short-term partial* loudness (STPL) is calculated by feeding the time series of IPL values into a temporal integration stage with an attack and a release time. The *long-term partial* loudness (LTPL) is calculated by feeding the time series of STPL values into another temporal integration stage with longer attack and release times than for STPL. The mean long-term loudness across the stimulus duration or its maximum were proposed to correspond to the overall loudness of a relatively long sound, such as a sentence or a musical phrase [37].

The loudness model was applied to the same set of experimental conditions and segment levels as in the experiment. In the experiment, a total of 38600 trials had been presented across the eight participants and the five experimental conditions. For each of these trials, a narrowband low-noise noise stimulus was generated using exactly the same sequence of 10 segment levels as in the corresponding trial of the experiment. This audio signal was fed into the loudness model, combined with a bandpass-filtered background noise of the same type and sound pressure level as in the corresponding trial, or without a background noise for the in-quiet conditions. To reduce the computation time, only 1 s of background noise was included before the onset of the stimulus. In the TVPL model, the long-term partial loudness is almost at its asymptotic value after 1 s, so that an inclusion of more than 1 s of background noise before signal onset would not have changed the simulation results. After stimulus offset, the background noise continued for 1.0 s, since in the experiment, the listeners typically responded within less than 1 s after target offset.

For each simulated trial, three statistics of STPL and LTPL were calculated. We selected the mean and the maximum because both were proposed to predict the overall perceived loudness [37]. We additionally included the 90^th^ percentile of both STPL and LTPL because some authors assume that percentiles may be preferable in estimating loudness [4,38].

In the loudness model, the STPL and LTPL do not return to 0 immediately after sound offset, but level off gradually across about 300 ms (STPL) and several seconds (LTPL) due to the release time constants. To account for these temporal dynamics, the three statistics were calculated across a temporal window starting at stimulus onset and ending 300 ms or 1000 ms after stimulus offset for STPL and LTPL, respectively. At 1000 ms after stimulus offset, LTPL is still at approximately 30% of its value before stimulus offset. However, in the experiment the next trial typically started after 1 s, so that a longer time window for analyzing the LTPL would not have been realistic. To summarize, the mean (STPL_mean_), the maximum (STPL_max_), and the 90^th^ percentile (STPL_pct_) of the short term partial loudness were calculated across a period of 1300 ms, and the mean (LTPL_mean_), maximum (LTPL_max_), and 90^th^ percentile (LTPL_pct_) of the long term partial loudness were calculated across a period of 2000 ms on each simulated trial.

**Fig 5:**
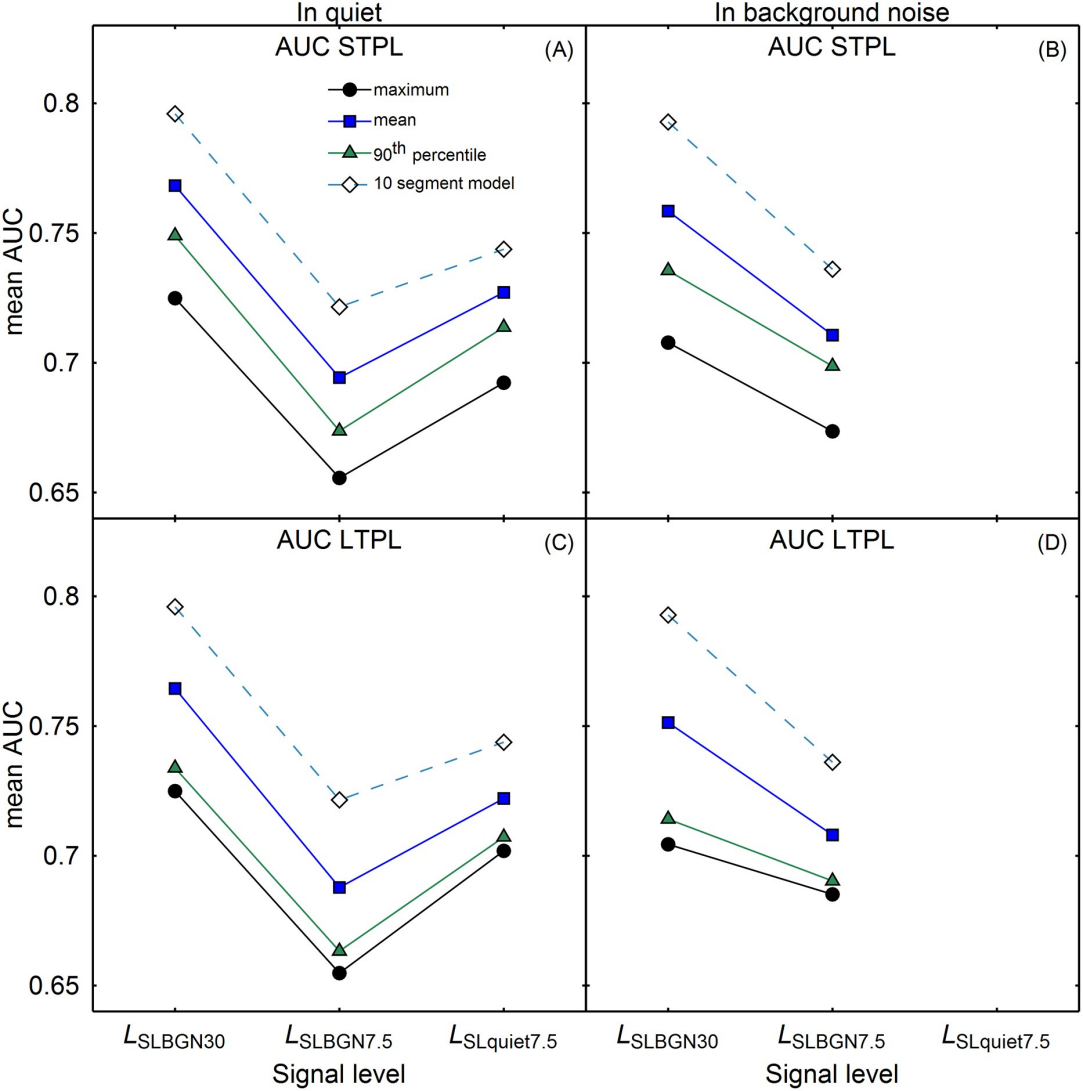
Mean AUC of the seven different regression models, as a function of signal level. Panels A and C on the left show the conditions in quiet, panels B and D on the right show the conditions in background noise. The two upper panels show the fits based on short-term partial loudness, the two lower panels show fits based on long-term partial loudness. The lines indicate the different summarizing statistics used as predictors (squares: mean; circles: maximum; triangles: 90^th^ percentile). The AUCs of the regression model based on the 10 segment levels (open diamonds) are plotted in each panel as a reference.

The a) ability of the loudness model to predict the responses of the listeners, and b) the temporal loudness weights predicted by the model were analyzed. Concerning a), separate logistic regression models were fitted for each combination of participant and experimental condition. For each simulated trial, the response of the participant in the experiment was used as the dependent variable, and a loudness statistic produced by the TVPL model (e.g., STPL_mean_) was entered as the predictor variable. The goodness-of-fit of the regression models based on the TVPL model were compared to the goodness-of-fit of the regression model based on the 10 segment levels that was used to analyze the data (see Fig 3). Fig 5 shows the resulting area under the ROC curve (AUC) for each of the regression models as an indicator of the goodness of fit. The regression based on the 10 segment levels provided the best fit to the data in quiet as well as in the presence of background noise at all signal levels. For a given TVPL-model loudness estimate (STPL, LTPL), the mean showed a better fit than the 90th percentile, which, in turn, showed a better fit than the maximum. This pattern was observed in each experimental condition. The AUCs for the conditions with the highest target level (BGN_SLBGN30_ & Quiet_SLBGN30_) were higher than in the conditions with lower target levels. However, in the in-quiet conditions the AUCs were higher in the condition Quiet_SLquiet7.5_ than in condition Quiet_SLBGN7.5_, although the target level in the former condition was higher.

An rmANOVA with the seven different regression models (mean, maximum, and 90^th^-percentile STPL; mean, maximum, and 90^th^-percentile LTPL; 10-segment model) and the five experimental conditions as within-subjects factors showed a significant main effect of model, *F*(6, 42) = 79.00, $\tilde{\varepsilon }$ = .311, *p* < .001, $\eta_{p}^{2}$ = .92. There was also a significant main effect of condition, *F*(4, 28) = 8.73, $\tilde{\varepsilon }$ = 1.0, *p* < .001, $\eta_{p}^{2}$ = .56. The model × condition interaction was significant but relatively weak, *F*(24, 168) = 4.90, $\tilde{\varepsilon }$ = .305, *p* < .001, $\eta_{p}^{2}$ = .41. As seen in Fig 5, the rank order of AUC across the seven models was identical in the five experimental conditions, but the exact difference between some models’ AUCs differed slightly as a function of experimental condition. Post-hoc pairwise comparisons were calculated between each of the TVPL-model based regression AUCs and the AUC of the regression based on the 10-segment model, averaged across experimental conditions. The AUC of the 10-segment model was significantly higher than the AUC of each of the TVPL-model based regression models (*p* < .01; Bonferroni-corrected). The minimum effect size in terms of Cohen’s *d*_z_ [39] was 2.91, which represents a strong effect.

**Fig 6:**
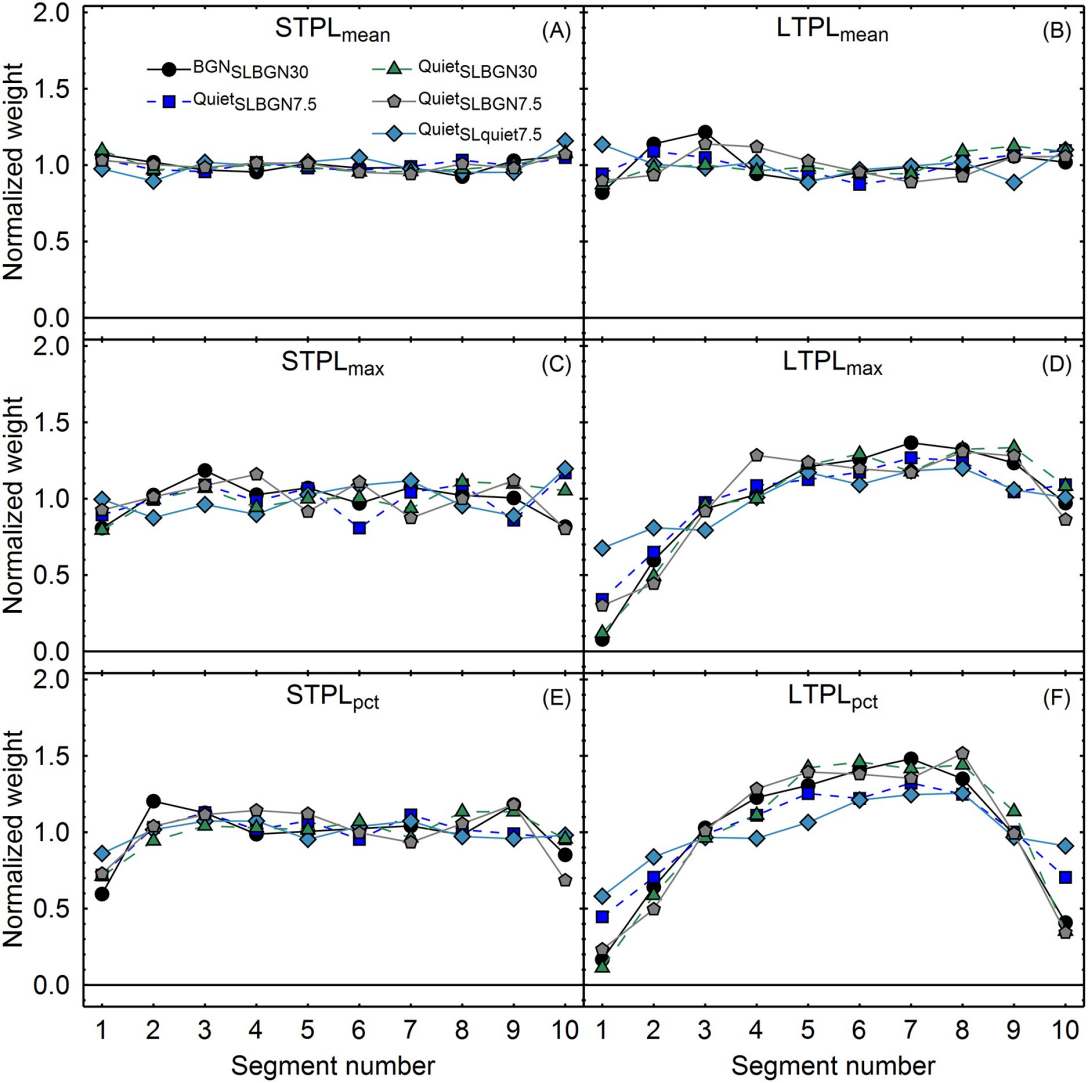
Mean normalized weights predicted by the TVPL model, as a function of segment number. The panels A-F show six combinations of summarizing statistic (mean, maximum and 90^th^ percentile; rows) and model loudness value (STPL and LTPL; columns). The lines indicate the five experimental conditions.

Concerning the temporal loudness weights predicted by the TVPL-model (b), each of the six loudness statistics produced by the TVPL model (e.g., STPL_mean_) were used to predict a response of either classifying the stimulus presented on the current trial as "louder" or classifying it as "softer", just as in the experiment. To this end, the given loudness statistic produced by the model (e.g., STPL_mean_) on a given trial was compared to the median of the same loudness statistic across all trials presenting the same experimental condition. If on a given trial the model loudness value was higher than this median value, the predicted response on this trial was "louder"; otherwise it was "softer". Next, exactly the same type of logistic regression model as we used for analyzing the psychoacoustic data was fitted for each of the five experimental conditions, except that the simulated response was used as the dependent variable rather than the observed response of the participants. Thus, the ten segment levels served as the predictors, and the simulated response served as the dependent variable. The regression coefficients were normalized as outlined in section Data analysis. Fig 6 shows the resulting normalized regression coefficients for each combination of summarizing statistic (mean, maximum, 90^th^ percentile), model loudness value (STPL, LTPL), and experimental condition. For each TVPL model statistic, the pattern of temporal weights was relatively stable across the different sound levels and not strongly affected by the presence or absence of a background noise, just as in the psychoacoustic data. However, none of the six TVPL model statistics predicted a pattern of temporal weights similar to the weights observed in the experiment (Fig 3). The mean STPL, maximum STPL, and mean LTPL predicted essentially flat temporal weighting profiles. The 90th percentile of the STPL predicts slightly lower weights on the beginning and end of the sound, while the maximum and the 90^th^ percentile of the LTPL predict an inverse U-shaped pattern with much lower weights on the beginning and/or end of the sound. Thus, none of the TVPL model statistics predicts the strong primacy effects observed in the experiment (see Fig 3).

### Conclusion

The present study showed that the primacy effect, i.e., the assignment of higher weights to the beginning of a sound when judging loudness, occurs even in the presence of a background noise. As the onset of the continuous background noise occurred well before the presentation of the to-be-judged target sounds, mechanisms such as attention orientation seem to be unlikely to be the source of the primacy effect. In addition, the size of the primacy effect was largely independent of the sound level. The parameters of an exponential decay function describing the time course of the primacy effect were comparable to those obtained in earlier studies, emphasizing that the time course of the primacy effect was hardly affected by the experimental manipulations. None of the estimates of the time-varying partial loudness model [34] could predict the obtained weights. Thus, further research appears necessary to gather a better understanding of the mechanism(s) underlying the primacy effect and its time course.
